# Use of Genetically Modified Mesenchymal Stem Cells to Treat Neurodegenerative Diseases

**DOI:** 10.3390/ijms15021719

**Published:** 2014-01-23

**Authors:** Robert D. Wyse, Gary L. Dunbar, Julien Rossignol

**Affiliations:** 1 Field Neurosciences Institute Laboratory for Restorative Neurology, Brain Research and Integrative Neuroscience Center, Program in Neuroscience, Central Michigan University, Mount Pleasant, MI 48859, USA; E-Mails: wyse1r@cmich.edu (R.D.W.); dunba1g@cmich.edu (G.L.D.); 2 Field Neurosciences Institute, Saginaw, MI 48604, USA; 3 College of Medicine, Central Michigan University, Mt. Pleasant, MI 48859, USA

**Keywords:** mesenchymal stem cells, genetic engineering, Parkinson’s, Alzheimer’s, Huntington’s, neurodegenerative diseases, transplantation, glial cell-derived neurotrophic factor (GDNF), brain derived neurotrophic factor **(**BDNF), nerve growth factor (NGF)

## Abstract

The transplantation of mesenchymal stem cells (MSCs) for treating neurodegenerative disorders has received growing attention recently because these cells are readily available, easily expanded in culture, and when transplanted, survive for relatively long periods of time. Given that such transplants have been shown to be safe in a variety of applications, in addition to recent findings that MSCs have useful immunomodulatory and chemotactic properties, the use of these cells as vehicles for delivering or producing beneficial proteins for therapeutic purposes has been the focus of several labs. In our lab, the use of genetic modified MSCs to release neurotrophic factors for the treatment of neurodegenerative diseases is of particular interest. Specifically, glial cell-derived neurotrophic factor (GDNF), nerve growth factor (NGF), and brain derived neurotrophic factor (BDNF) have been recognized as therapeutic trophic factors for Parkinson’s, Alzheimer’s and Huntington’s diseases, respectively. The aim of this literature review is to provide insights into: (1) the inherent properties of MSCs as a platform for neurotrophic factor delivery; (2) the molecular tools available for genetic manipulation of MSCs; (3) the rationale for utilizing various neurotrophic factors for particular neurodegenerative diseases; and (4) the clinical challenges of utilizing genetically modified MSCs.

## 1. Aims of Review

This review provides information on the current developments and challenges of MSCs (mesenchymal stem cells) as a cell-based intervention for neurodegenerative diseases, in particular Parkinson’s, Alzheimer’s and Huntington’s disease. Additionally, rationale for the use of MSCs as a biological device to deliver therapeutic molecules targeting disease pathology is provided. Furthermore, the molecular methods to modify MSCs for the enhancement of their therapeutic utility are discussed in detail.

## 2. MSC Background

Derived from a wide range of sources, MSCs are capable of long-term survival and replication in culture [1]. The primary sources utilized from which of MSCs are derived include bone marrow (BM), adipose tissue (ADI) [2], and umbilical cord blood (UCB) [3]. Till and McCulloch [4] have described the *in vivo* clonal nature of bone marrow cells, while Friedenstein and colleagues [5] provided an *in vitro* assay to evaluate the clonogenic potential of these cells, identifying them as colony-forming units-fibroblastics (CFU-Fs). A standardized set of criteria to define MSCs was set forth by The International Society for Cellular Therapy in an attempt to standardize MSC nomenclature. These criteria mandate that the MSCs be plastic adherent, express CD105, CD73 and CD90, while lacking CD45, CD34, CD14, CD11b, CD79α, CD19, or human leukocyte antigen (HLA) DR expression. In addition, MSCs must differentiate into osteoblasts, adipocytes and chondroblasts *in vitro* [6]. Although these criteria are generally accepted, a variety of factors, such as source of the cell [1], isolation protocols [7], culturing methods [8], and lack of a specific marker [9], create a challenge to define MSC unambiguously. The title of MSCs, which was popularized by Caplan [10], has become rather nebulous ensuing a debate on the appropriate use of the identifiers, stem or stromal, in the title [11]. With the indistinctive title of MSC many laboratories have assigned different names for their preparations, such as multipotent adult progenitor cells [12], unrestricted somatic stem cells [13], and multidifferentiated mesenchymal progenitor cells [14] as a means to appropriate title cell preparations. Use of different isolation methods and culturing techniques give rise to a variety of cell populations with unique characteristics [15]. In order to make accurate comparisons of the efficacy of the therapeutic uses of MSCS, further standardization that specifies the reporting of phenotypic cell markers and genetic expression profiles are needed.

With the challenge of standardization aside, MSCs serve as readily accessible cell populations that are easily amplified [16] and contain several beneficial capabilities. The low immunogenicity and immunomodulatory capacity of MSCs may be seen as the most valuable features of these cells. The immunomodulatory effect of transplanted MSCs is most apparent in the treatment of graft *versus* host disease [17–19]. The exact mechanisms of immunomodulation are currently unknown, but a large repository of evidence [20] suggests that, through an interferon-γ initiated pathway [21], MSCs can secrete indoleamine 2,3-dioxygenase and prostaglandin E2 [22], leading to the suppression of both T-cell [23] and natural killer cell proliferation.

The chemotaxic properties of MSCs have gained attention recently, as MSCs have been observed to migrate through the internal environment towards sites of inflammation [24]. The homing responses of MSCs are directed by a host of chemokines and growth factors and can be harnessed and enhanced through pre-exposure to inflammatory cytokines [25] or genetic modification, prior to transplantation. One signaling system that has been utilized for this purpose is the signaling factor stromal cell-derived factor-1 (SDF-1), which is expressed in areas of inflammation in the brain [26,27]. When the chemokine receptor type 4 (CXCR4), which responds to SDF-1, is overexpressed in MSCs, it increases homing functions for disease-specific areas related to acute kidney injury [28], myocardial infarction [29], glioblastoma [30], and ischemic stroke [31]. This homing system has been successfully used in other studies without direct genetic overexpression of chemokine receptors produced by MSC pre-conditioning, maintenance in hypoxic conditions (low O_2_, 5%), or treatment with factors that mimic hypoxia [32]. The up-regulation of receptors in MSCs through hypoxic exposure has been related to an increase in therapeutic efficacy following systemic [33] or intranasal [34] administration in animal models ischemic stroke. MSCs that were maintained in a hypoxic environment had a higher migration response *in vitro* to growth factors, chemokines, and inflammatory cytokines, compared to MSCs maintained in normoxic conditions [35]. The hypoxic maintenance of MSCs provides a valuable tool to increase the homing capacity of MSCs as a therapeutic intervention. However, the homing capacity of MSCs is diminished through extended passaging [36], or when cultured beyond confluency [37].

A rising concern with the therapeutic use of MSCs is the mode of delivery. Through chemotaxis, systemic delivery has shown promise in the ability of MSCs to home in on areas of injury [38]. However, there is often a wide dispersal of MSCs throughout many other organs beyond the area of injury. Generally, administration into the peripheral venous system results in an initial concentration of MSCs in the lungs, followed by a gradual targeting of the injured area [39]. However, combinatory treatment of heparin with MSCs injection has been shown to circumvent the initial concentration of cells in the lungs following inter-venous injection [40], and an intra-arterial injection close to the site of injury has been shown to increase homing efficacy [41]. While intra-arterial injections may increase the number of engrafted cells, this delivery method also generates an enhanced risk of vascular occlusion [41]. The method of delivery, local, intra-arterial, or intravenous should be tailored to the goals of the therapeutic intervention while considering the potential risks of each method.

Although systemic delivery of MSCs is noninvasive, the blood brain barrier (BBB) presents a significant challenge to effective treatment in central nervous system disorders [42]. Several studies have shown that MSCs were not able to cross the BBB following systemic delivery [43,44] due to a variety of challenges [45]. However, there is growing evidence that MSCs may be able to cross the BBB in sufficient numbers to elicit a therapeutic benefit [42,45]. For example, ADI MSCs have been shown to cross the BBB following intravenous injection in a mouse model of Alzheimer’s disease [46]. In addition, intra-arterially delivered BM mononuclear cells migrated into the brain of ischemic stroke patients within two hours following their administration [47]. This ability for MSCs to cross the BBB following systemic delivery has been observed in several other studies [42,48], suggesting that systemic delivery of MSCs may be a feasible method of delivery. While observations of MSCs crossing the BBB have been made, this ability may be dependent upon disruption or dysfunction of the BBB [46]. Additionally, the state of BBB function, through the progression of a disease or injury [49] may impact the therapeutic efficacy of systemic administrated MSCs.

Upon reaching the area of interest, MSCs seem to release factors that ameliorate the degenerating or diseased environment. The paracrine signaling of transplanted MSCs has been cited as the primary mechanism of recovery in a wide range of studies [50]. As discussed above, MSCs have been shown to possess immunomodulatory functions and secrete a wide range of neuroprotective proteins [51]. It has been observed that MSCs can secrete brain derived neurotrophic factor (BDNF), nerve growth factor (NGF), vascular endothelial growth factor (VEGF), and hepatocyte growth factor (HGF) [52]. Further analyses have revealed that sub-populations of MSCs express higher levels of such factors [53]. These beneficial factors secreted by MSCs (since termed the secretome) can be observed when injecting conditioned medium, but only when the fraction is greater than 10 kDa, as observed in a model of spinal cord injury [54] and myocardial infarction in mice [55] and pigs [56]. These studies highly implicate the role of MSCs in paracrine signaling, and provide an opportunity for experimenters to develop new methods of genetically enhancing the therapeutic function of MSCs.

## 3. Genetic Delivery Using MSCs

Recently, a significant attention has been placed on the therapeutic potential of extracellular vesicles, in particular exosomes, that are approximately 30–200 nm in size [57]. These exosomes were originally considered solely responsible for the removal of aberrant materials [58], but now have been shown to carry a variety of critical molecules, including mRNA, miRNA proteins, lipids, mitochondrial DNA, and genomic DNA [59,60]. Exosomes are formed by the invagination of the endosomal membrane, released after fusion of multivesicular bodies with the plasma membrane [61] and are involved in cell-to-cell communication in a wide variety of cells and systems [62]. The endosomal signaling of MSCs has been shown in multiple studies to be at least partially responsible for the therapeutic effect of transplanted MSCs [63], thus further implicating the therapeutic role of signaling from MSCs.

Researchers have utilized the secretome of MSCs in order to deliver therapeutic molecules in non-invasive manners that can target areas of degeneration. The endogenous incorporation of a particular protein into exosomes can be guided through chimeric infusion of the protein of interest to the C1C2 domain of MFG-E8/lactadherin protein in place of the EGF domain [64]. This technique results in an exosome-bound protein that has been shown to be more effective than a soluble counterpart in the reduction of tumor size, resulting from a directed immune response [65]. This technique has also been used to specifically target the brain by replacing the EGF domain with the rabies viral glycoprotein (RVG) peptide resulting in systemically administered MSC-derived exosomes that cross the BBB. These exosomes then deliver exogenously loaded Beta-secretase 1 (BACE1) siRNA, which can knock down BACE1 (Alzheimer’s disease related protein), mRNA, and protein levels in neurons, microglia and oligodendrocytes [66].

Overexpression of a therapeutic gene has also been shown to result in exosomal-mediated transfer of the therapeutic mRNA and protein to the desired target [67]. While general overexpression is not precise, specific targeting of RNA to exosomes may be possible through the modification of the 3′-UTR of a therapeutic sequence to include a 25 nucleotide segment that contains a binding site for microRNA-1289, which in turn mediates the packaging of mRNA into exosomes [68].

Pretreatment of MSCs under specific conditions, has been shown to modify the secretome of MSCs and change their efficacy in treatment. Xin and colleagues [69] observed the efficacy of pre-treating MSCs with ischemic brain extracts. The pre-treated MSCs showed an increased expression of microRNA-133b that was able to be transferred to neurons and astrocytes through exosomes, resulting in an increase of neurite length and branching of treated neurons [69].

Through genetic manipulation the basal therapeutic immunomodulatory, chemotaxic, and signaling functions of MSCs can be enhanced in order to develop a specific therapy designed to target a particular disease mechanism. A variety of methods are available to introduce exogenous genes to be expressed by MSCs. The most frequently utilized method is the viral vector.

### 3.1. Lentivirus & Retrovirus

A major advantage of using MSCs for stem cell therapies is the ease at which they can be manipulated, either through changes in culture conditions or virally mediated genetic engineering. Lentivirus- and retrovirus-based delivery systems are efficient and are capable of integrating into the genome ensuring the long-term expression of the transgene [70]. Retroviral transfection of the deficient IL-2 receptor γ (IL2RG) in autologous hematopoietic stem cells, *ex vivo*, has been clinically utilized in the treatment of X-linked severe combined immunodeficiency (SCID) [71] and the treatment of other diseases [72]. However, it was observed in a recent study that the use of the retrovirus vector as led to insertional mutagenesis, resulting in activation of proto-oncogenes in four of fifteen patients [73]. The lentivirus, which is part of the retrovirus family is capable of transfecting dividing and non-dividing cells [74]. Clinical success utilizing a lentiviral vector has been observed in the treatment of X-linked adrenoleukodystrophy in the same fashion as X-linked SCID [75], but without any reported complications. Lentiviral gene therapy is generally accepted to be safer than use of a retrovirus because of the measures taken to remove viral components and the extensive pre-clinical testing that has been conducted [76]. Although the use of the lentiviral vector causes fewer insertional mutations, this procedure is not without risk. Bokhoven and colleagues [77] developed a cell culture assay to identify insertional mutagenesis in key genes and found no differences between the rate of mutagenesis of a retrovirus and a lentivirus, albeit these were mediated through different mechanisms [77].

### 3.2. Adenovirus

Adenoviruses do not integrate into the host genome and can infect both dividing and non-dividing cells, with transient expression in dividing cells and long-term expression in non-dividing cells. First generation adenoviruses are highly immunogenic, and in one case, has led to the fatal immune response of a patient treated for ornithine transcarbamylase deficiency [78]. Adeno-associated viruses (AAVs) have a lower immunogenicity and are the first vector approved for gene therapy in the Western world, designed to treat lipoprotein lipase deficiency [79]. Thus, AAV-based gene delivery is generally considered to be a safer alternative then lentiviral and retroviral gene delivery, primarily due to its lack of integration, although problems of immunogenicity are still of concern [80]. While the AAV does not functionally insert the gene into the genome, random homologous and heterologous recombination into the host genome can occur [81]. Even though transfection of MSCs with adenoviruses or AAVs does not lead to prolonged gene expression, intra-arterially administered human BM-MSCs that were transduced to express interferon-β (IFN-β) via an adenovirus had sufficiently lasting effects to increase the survival time of rats with induced glioblastomas [82]. Subsequently, MSCs have also been utilized as a vector for delivery of adenovirus in multiple glioblastoma studies [83,84].

### 3.3. Non-Viral Agents

Non-viral mediation of gene transduction into MSCs is an increasingly attractive avenue for gene therapy, because these methods circumvent complications of viral-based gene insertion. Alternative techniques include electroporation [85], lipofection [86], nucleofection [87], as well as the use of calcium phosphate nanoparticles [88], nanoneedles [89], and polysaccharides [90–93]. The source of MSCs has been shown to influence the efficiency of transduction [94], so the use of these procedures necessitates appropriate testing and investigation into which cell type is most effective for non-viral gene delivery.

Zinc finger nuclease (ZFN) [95], transcription activator-like effector nuclease (TALEN) [96], and the CRISPR/Cas system [97] are new tools for genetic modification. Each of these tools can be designed to safely target a specific sequence in determined loci and initiate a double strand break of DNA, initiation endogenous DNA repair, which allows for the insertion of the therapeutic gene [98]. Intraperitoneal administration of human BM, UCB, and ADI MSCs resulted in relatively long-term elevation of erythropoietin plasma levels when ZFN methods were utilized to overexpress the erythropoietin gene [99]. These methods have rarely been investigated with MSCs, but may prove to be an invaluable resource for genetic engineering, as they are being increasingly utilized in many other applications [98].

Development of human artificial chromosomes (HACs) is another promising avenue of non-viral mediated transgene expression in MSCs, due to their proven stability. Through truncation of the 21st chromosome and introduction of a Cre-loxP gene insertion target, HACs capitalize upon the cell cycle for stable expression, reduce the risk of aberrant integration, offer control over copy number, and can carry an extremely large payload (of up to 10 Mb), allowing for entire genes to be transduced [100]. Hoshiya and colleagues [101] observed that HACs, carrying the entire dystrophin gene (of about 2.4 Mb, including both coding and regulatory portions), were stable in human immortalized MSCs for 100 population doublings. Stable expression of transgenes in MSCs with HACs has been verified in other studies as well [102,103]. Additionally, Kinoshita and colleagues [104] observed, in a rat model of glioma, that an injection of human MSCs, transduced with a HAC carrying the thymidine kinase gene, as well as an infusion of ganciclovir, resulted in the reduction of glioma volume.

The US Food and Drug Administration does not currently approve gene therapy for the general public, as there have been several clinical challenges when using this method [105]. The pursuit of safe gene therapy vectors and treatment of a wide range of diseases through *ex vivo* manipulation of the patient cells and re-administration is encouraged by Szybalski [106], who originally demonstrated genetic transformation.

## 4. Genetically Engineering MSCs for Parkinson’s Disease

Primary symptoms of Parkinson’s disease (PD) include asymmetric resting tremor, rigidity, and bradykinesia [107]. Ten percent of those diagnosed with Parkinson’s disease are of the familial type, which is associated with multiple mutations in several genes [108]. The age of onset, which can be influenced by familial history, usually occurs during the third or fourth decade of life [108]. Degeneration of dopaminergic neurons in the substantia nigra is the identified cause of symptoms, which do not arise until fifty percent of nigral neurons are lost and there is an eighty percent decrease of striatal dopamine [109]. The dysfunctions of mitochondrial activity, inflammation, oxidative stress, and protein misfolding have all been identified as antecedents of PD pathology. However, these changes may be the result of other downstream effects that might be the primary cause of PD [110]. Intraneuronal Lewy bodies, containing alpha-synuclein aggregation, are found throughout the brain and are pathological hallmarks of PD [111]. The most frequent treatment for PD is levodopa (L-DOPA), which increases dopamine levels, leading to improved motor function, but this therapy frequently results in L-DOPA-induced dyskinesia [112]. Surgical ablation of targets within the thalamus and pallidus were utilized to treat PD symptoms in the past, but deep brain stimulation (DBS) is the currently preferred therapy [113]. While DBS is intrusive, as electrodes must be placed in the same target areas used in ablation techniques, a benefit is that the electrodes can be adjusted to provide the most efficacious treatment for the patient [113]. However the current treatments for PD only mitigate the symptoms and do not treat the underlying pathology, hence the need for alternative therapies is evident [114].

Use of glial derived neurotrophic factor (GDNF) was first identified by Lin and colleagues [115] as a potential treatment for PD because of its ability to increase the dopamine uptake in, and the survival of, embryonic ventral midbrain dopaminergic neurons [116]. The mechanisms of GDNF protection are still unknown, but the GDNF family receptor-1α signal is transduced by Ret, a membrane receptor protein tyrosine kinase [117]. There are three additional paralogs of the GDNF ligand and receptor genes in humans, which have been investigated for their therapeutic value, along with multiple orthologs in other vertebrates [118]. Ret stimulation, via GDNF, cascades to activate the PI3K/Akt and MAPK/Erk pathways [119]. Activation of the PI3K/Akt pathway has been implicated in GDNF-mediated neuronal survival [120,121]. Given that ninety-five percent of GDNF-expressing neurons in the striatum are parvalbumin-positive interneurons [122], targeting means of activating endogenous populations of these cell types or culturing them for subsequent transplantation may serve as an effective means for potential treating PD.

Clinical trials using GDNF for treating PD have produced mixed results. A double-blind study, in which a range of 25–4,000 micrograms of GDNF was delivered via a catheter into the right ventricle of PD patients did not yield improvements on the Unified Parkinson’s Disease Rating Scale (UPDRS); in addition, several side effects were initially observed, but were subsequently resolved following the discontinuation of treatment [123]. Another trial utilized bilateral, direct pump infusion of GDNF to the putamen of PD patients, and yielded a 39% decrease in off-medication UPDRS scores, a 64% reduction of L-DOPA induced dyskinesias, and a 28% increase of dopamine in the putamen 18 months following treatment, as measured by [^18^F]dopa positron emission tomography [124]. In addition, the unilateral infusion of GDNF into the putamen of ten PD patients proved equally beneficial for at least six months [125]. It is important to note that the successful trials using infusion of GDNF into the putamen were open-label, while the double-blind study did not yield positive outcomes [126]. However, the lack of beneficial results in double-blind studies may have been the result of inadequate diffusion of GDNF [127], rather than experiment bias on placebo effects.

With mixed clinical results and challenges of infusion, *ex vivo* cell-based strategies to deliver GDNF has gained recent attention as an appropriate alternative [128]. As shown in Table 1, use of MSCs for delivering trophic factors for treating PD and other neurodegenerative diseases has provided promising results. An early pilot study, conducted by Venkataramana and colleagues [129], in which autologous BM-MSCs were injected unilaterally into the lateral wall of the lateral ventricle in seven PD patients (one million cells per kg), yielded a 22.9% for off-drug and a 31.7% for on-drug improvement of UPDRS scores in three of the seven patients, and while producing no adverse events. This study was extended by bilaterally injecting a dose of two million BM-MSCs per kg from healthy donors into twelve PD (8 early-, 4 late-onset) patients, resulting in UPDRS score improvement of 17.92% for on-drug and 31.21% for off-drug in early-onset patients, with no improvements identified in late-onset patients [130]. The positive therapeutic results and the lack of serious adverse responses during these pilot studies, suggests that treatments using MSCs may have significant clinical utility for PD.

The engineering of MSCs to express GDNF has received much attention recently. MSCs transduced with a retrovirus to express GDNF increased dopaminergic neuron sprouting when transplanted in the striatum at four days prior to 6-OHDA injection in a rat model of PD [131]. Glavaski-Joksimovic and colleagues [132] observed a decrease in amphetamine-induced rotations following a unilateral transplantation of human MSCs (expressing GDNF and transiently pre-differentiated with a Notch plasmid) into the striatum at one week following unilateral 6-OHDA administration into the same rat striatum. In another study, Sadan and colleagues [133] transplanted human MSCs primed with mitogens (basic fibroblast growth factor, epidermal growth factor, and platelet derived growth factor) that had a fivefold increase of BDNF and twofold increase of GDNF expression compared to unprimed MSCs and were more efficacious than unprimed MSCs in ameliorating behavioral and dopamine deficits in a rat striatal 6-OHDA lesion model. Using another approach, intrastriatal injections of MSCs, transduced with a lentivirus to overexpress GDNF, were given one week before a lactacystin lesion of the medial forebrain bundle and significantly increased striatal dopamine levels, while reducing apomorphine-induced rotations [134]. Furthermore, Ren and colleagues [135] transplanted autologous BM-MSCs, that were genetically modified to overexpress GDNF, into the striatum and substantia nigra of MPTP-treated non-human primates. The transplants of these cells increased levels of dopamine in the striatum and improved contralateral limb function, but did not prevent the loss of nigral dopaminergic neurons.

## 5. Genetically Engineering MSCs for Alzheimer’s Disease

The primary symptoms of AD are cognitive dysfunction and dementia, which include a wide range of other distressing emotional and behavioral concerns [141]. An estimated 35.6 million people were affected with Alzheimer’s disease (AD) worldwide in 2010 [142] and the prevalence is expected to quadruple by 2050, with 1 in 85 people living with AD [143]. The Alzheimer’s Association currently estimates that 5.4 million Americans have AD [144].

Investigations into familial AD have identified dominant mutations of the amyloid precursor protein (APP), SorL1, and presenilin 1 and 2. These mutations account for five percent of AD diagnoses [145]. Several other genes have been identified as increasing the risk of AD [146], especially the epsilon 4 allele of the apolipoprotein E (APOE) gene. Ninety-one percent of homozygous carriers for APOEɛ4 develop AD at a mean onset of 68 years of age, 47% of heterozygotes at 76 years, and 20% for non-carriers at 84 years [147]. The AD-related genes directly or indirectly interact with the function of APP and its cleaved constituents, amyloid beta 1–40 (Aβ_1–40_) and Aβ_1–42_, or with the tau protein, which in their aggregated forms, produce amyloid plaques [148] and neurofibrillary tangles [149], the characteristic hallmarks of AD. It is uncertain if plaques and tangles are causative or protective, but they are observed in conjunction with glial responses, as well as neuron and synaptic loss. Plaques are generally found throughout the cortex and tangles are thought to progress from the entorhinal cortex, to the hippocampal subfields and then spread to the amygdala, association cortex, and throughout the neocortex, respectively [150]. Observations of a reduction of neurons in the basal forebrain cholinergic nuclei (BFChN) and reduced levels of choline acetyltransferase, acetylcholine, and acetylcholinesterase (AChE) in the hippocampus and neocortex led to the development of the cholinergic hypothesis of AD [151]. The Food and Drug Association (FDA) has approved AChE inhibitors for treatment of mild to moderate AD. Memantine, a *N*-methyl-d-aspartate receptor agonist, has also been approved for treatment of moderate to severe AD [152]. These pharmacological interventions have been shown to provide only palliative benefits, partially delaying the progression of the disease, but are generally well tolerated [153].

However, because current AD treatments focus on symptoms related to neurotransmitter systems and do not target underlying pathologies, there is a clear need for more targeted interventions [154]. In attempts to focus on disease pathology, a variety of small molecule inhibitors have been identified that minimize the fibril formation of Aβ [155] and tau [156], but these drugs have faced many challenges in clinical trials [157]. Similar attempts with immunotherapy, targeted at plaques and tangles, are underway. This approach is targeted to diffuse aggregates, but overarching concerns of developing autoimmunity still need to be addressed [158]. Given the widespread prevalence of the disease and the lack of safe treatments that target the pathology, the development of new therapeutic strategies for AD is a critical need.

One such strategy involves the use of, nerve growth factor (NGF). Given that reduced retrograde transport of NGF from the hippocampus to the BFChN in AD patients was reported by Mufson, Conner, and Kordower [159], much attention has been given to modulating two levels of this molecules as a potential therapy for AD. NGF has a high affinity for tropomyosin kinase receptor A (TrkA), but a low affinity for the non-selective neurotrophin receptor p75 (P75NTR) [160]. TrkA signaling promotes survival, primarily through Ras activation of the PI3K\AKT pathway, although there are many other identified signaling pathways [161]. NGF signaling is mediated by TrkA and P75NTR interactions, whereby both pro-survival or apoptotic-regulatory signaling can occur [162]. The relationship of these receptors is important in AD pathology, as a reduction of TrkA, but not P75NTR, in BFChN neurons leads to increased behavioral symptomology [163,164] and Aβ pathology [165]. These receptor relationships develop a time course of AD pathology, whereby NGF serves as a protective molecule at first, but then engages in degenerative responses [166]. With evidence of recovery in pre-clinical models [167,168], and an increasing understanding of the variables involved in the relationship between NGF and AD pathology [169], there is growing interest in the efficacy of NGF for treating AD [170].

Due to the inability of NGF to cross the BBB [171], initial trials were conducted using direct administration in the brain. In an early single-subject study, an AD patient was given intraventricular administration of murine recombinant NGF over three months which resulted in improvement of verbal episodic memory, but also caused significant weight loss [172]. Side effects, including back pain and weight loss, were observed in a similar trial of three AD patients, which was terminated early because no noticeable clinical improvements were apparent [173]. The pain response following NGF treatment is a result of the role NGF plays in the inflammatory response [174]. The nociceptive challenge of NGF treatment is being addressed by development of mutant NGF proteins that selectively activate TrkA receptors, but not P75NTR. These mutant NGF proteins may lead to an increased therapeutic potential via their agonist effects on TrkA receptors, without the concomitant pain and other adverse effects with P75NTR activation [175,176].

While direct infusion of NGF has had complications, a clinical trial of eight AD patients utilizing autologous fibroblasts, engineered to express NGF, were transplanted into the nucleus basalis of Meynert which produced NGF expression in grafts, a reduced decline or improvement of Mini-Mental Status Examination scores, a reduced decline of the Alzheimer Disease Assessment Scale-Cognitive subcomponent, and an increase in glucose activity in several areas receiving cholengergic input from transplant site [177]. These improvements were observed without adverse reactions to the NGF for twenty two months [177]. A phase II clinical trial of this method is currently open [178]. The treatment of AD with MSCs has not been studied directly in clinical trials, but results of some pre-clinical research are available.

Transplants of BM-MSCs have been shown to have considerable promise for treating AD (see Table 1). Transplants of BM-MSCs have been reported to reduce Aβ through microglial activation in an acute Aβ-induced mouse model of AD [136]. Lee and colleagues [137] transplanted human UCB-MSCs at three different time periods into the hippocampus of a transgenic mouse model of AD, observing improvements in the Morris-water-maze task and reductions in Aβ and tau aggregation, presumably mediated through immunomodulation.

A proposed mechanism of this UCB-MSC mediated protection is through release of soluble intracellular adhesion molecule-1 which increases neprilysin, an Aβ-degrading enzyme, in microglia [179]. Using another source of MSCs, Katsuda and colleagues [180] also identified that ADI-MSCs directly secrete neprilysin. Following their observations of a therapeutic effect of BM-MSC when transplanted into the hippocampus of an acute Aβ-induced rat model [138], Wu and colleagues [139] used lipofection techniques to overexpress NGF in MSCs. These investigations observed an enhanced therapeutic effect of the NGF-transduced MSCs, transplanted in the same manner, to reduced memory deficits in the Morris-water-maze task.

## 6. Genetically Engineering MSCs for Huntington’s Disease

The primary characteristics of Huntington’s disease (HD) are choreic movement, along with emotional and cognitive disturbances, which generally develop at forty to fifty years of age, progressing for ten to twenty years, eventually resulting in death [181]. Prevalence of HD is 5.70 per 100,000 in Europe, North American, and Australia [182]. The cause of HD is a heritable autosomal dominant mutation, that exists as a polyglutamine (CAG) repeat within the first exon of the IT15 gene on the fourth chromosome within region p16.3 [183]. The disease has been identified to progress in conjunction with degeneration of medium spiny neurons within the striatum, specifically the caudate nucleus and putamen [184]. HD symptomology is observed when CAG repeats are greater than 36 and genetic anticipation has been associated with paternal transmission, with increased instability above 46 repeats [185]. The direct role of the huntingtin protein is still unknown, but the CAG repeat loci is an important feature, as this protein contains huntingtin, EFP3, PP2A, and TOR1 (HEAT) consensus areas, suggesting that the it is highly involved in protein-to-protein interactions, given that other proteins possessing a HEAT complex have been identified to be involved with regulatory cytoplasmic properties in eukaryotic cells [186]. In addition to the HEAT complexes, there are several protease cleavage sites, which are believed to lead to the toxic fragments [187], found as intranuclear inclusions and cytoplasmic aggregates. These aggregates are hallmarks of HD and have been shown to correlate with the progressive neurodegeneration [188]. A large body of evidence suggests that the HD aggregates reflect deficits in the ubiquitin-proteasome system, given toxic aggregates are labeled with ubiquitin, but are not sufficiently cleared [189]. However, there is many other hypotheses, including excitotoxicity, proteolysis, protein misfolding, autophagy, and mitochondrial dysfunctions, have also been implicated in the pathology of HD [190].

One characteristic of HD, that has received considerable attention recently is that reduced levels of BDNF are found in the striatum of HD patients [191]. BDNF is anterogradely transported to the striatum primarily from the neocortex but also from the pars compacta, amygdala, and central medial thalamic nucleus [192]. The lower BDNF levels in the striatum are partially due to a loss of function of the wild-type huntingtin protein, that inhibits repressor element 1/neuron-restrictive silencer element (RE1/NSRE), which, in turn, silences the BDNF exon II promoter [193]. In addition to transcriptional influences on BDNF, the mutant huntingtin protein adversely affects proper BDNF transport and secretion, as wild-type huntingtin assists with the vesicle transport of BDNF along microtubules by forming a complex with huntingtin-associated protein I and the p150Glued subunit of dynactin [193].

Currently, there is no cure for HD, and only palliative treatments are available. In the past 10 years, several drugs have been investigated in clinical trials with no new approvals [194]. Treatment focuses on symptoms [195] and the only therapy currently available for HD is tetrabenazine, which selectively depletes monoamines particularly in the striatum resulting in less movement-related symptoms [196]. Pre-clinical investigations have shown that the use of small-interfering RNAs (siRNAs) which reduce the levels of both mutant and wild-type huntingtin, have, nonetheless, been shown to reduce histological and behavioral deficits in rodents [197–199] and in non-human primates [200,201]. MSCs transduced with a lentivirus to express siRNA targeted at mutant huntingtin has provided encouraging results, *in vitro*, as 50% of mutant huntingtin is knocked down in HD affected neurons, but technical challenges have prevented a robust *in vivo* effect [202].

Exogenous administration of BDNF is another area being explored in the development of HD therapies. BDNF, which is an agonist for tropomyosin kinase receptor B (TrkB), is important in synaptic plasticity [203] and neuronal survival [204]. BDNF has been shown to provide trophic support and influence differentiation of striatal GABAergic neurons *in vitro* [205,206]. Administration of BDNF to rat neuronal cultures containing mutant huntingtin is neuroprotective [207]. In addition, partial striatal protection has been observed *in vivo*, when BDNF was delivered directly to the striatum of R6/1 mice [208].

Work in our lab [140] has shown that BM-MSCs that are modified using a retrovirus to overexpress NGF or BDNF, can produce a 6.8-fold increase in BDNF production and 4.6-fold increase in NGF over non-transfected MSCs. The genetically modified MSCs were then bilaterally transplanted into the striatum of YAC mice at four months of age. Behaviorally, clasping was reduced in all groups that received MSCs, regardless of the cell type, and deficits on the rotorod task were ameliorated in the group receiving BDNF-modified MSCs. Striatal NeuN positive cell counts were restored to wild-type levels in BDNF-MSC treated mice. Our preclinical findings clearly demonstrate that MSCs, transduced with BDNF, provide behavioral and histological improvement over MSCs that have not been genetically engineered. From these findings, Wheelock and colleagues [209] are expanding this research and beginning clinical trials utilizing genetically engineered human MSCs that overexpress BDNF for treatment of HD. The pre-cellular therapy observation study is currently enrolling [210].

## 7. Conclusions

The history of both clinical and pre-clinical trials reveal that MSCs provide considerable promise as therapeutic agents [211–213], especially as sources for producing or vectors for delivering neurotrophic factors [214] for the treatment of neurodegenerative diseases (see Table 1).

Research into developing safe methods to deliver therapeutic MSCs [215] should be a priority, as complications and mortality have plagued previous attempts at intracranial transplantations of therapeutic cells [177]. To maximize the potential therapeutic benefit, attention must be placed on the culturing conditions of MSCs prior to transplantation, as these factors greatly affect the utility and safety of MSCs [216,217]. Continued investigation into the use of various tissue sources, approaches to genetic manipulation, methods of administration, and clinical challenges facing therapeutic MSCs are needed to facilitate the development of new therapies neurodegenerative diseases.

## Figures and Tables

**Table 1.** Selected studies utilizing MSCs for the treatment of neurodegenerative diseases.

Disease	Species, Model	Note
PD	Human	Symptom reduction with no adverse effects following unilateral BM-MSC transplant [129].
PD	Human	Improvement in early-stage patients following bilateral BM-MSC transplant [130].
PD	Rat, Intrastriatal 6-OHDA	GDNF-BM-MSCs enhanced tyrosine hydroxylase labeling and dopaminergic neuronal sprouting in striatum [131].
PD	Rat, Intrastriatal 6-OHDA	GDNF-Notch-BM-MSCs decreased amphetamine-induced rotation [132].
PD	Rat, Intrastriatal 6-OHDA	Mitogen treated human BM-MSCs reduced behavioral and dopamine deficits [133].
PD	Rat, Medial forebrain bundle lactacystin lesion	GDNF-BM-MSCs increased striatal dopamine levels and reduced apomorphine-induced rotation [134].
PD	Non-human primate, MPTP	Unilateral transplant of GDNF-BM-MSCs into striatum and substantia nigra increased contralateral motor performance and increased dopamine levels at area of transplant [135].
AD	Mouse, Acute amyloid-β aggregate	Levels of amyloid-β were reduced following BM-MSC administration [136].
AD	Mouse, Acute amyloid-β aggregate	Delivery of human UCB-MSCs reduced behavioral deficits and toxic aggregation presumably through immunomodulation [137].
AD	Rat, Acute amyloid-β aggregate	NGF-BM-MSCs provided enhanced therapeutic effects over non treated BM-MSCs [138,139].
HD	Mouse, YAC128	BDNF-MSCs ameliorated rotorod deficits and restored striatal neuronal loss [140].
